# Function and dysfunction of presenilin in Alzheimer’s disease

**DOI:** 10.1186/1750-1326-8-S1-O11

**Published:** 2013-09-13

**Authors:** Jie Shen

**Affiliations:** 1 Harvard Medical School, Boston, USA

## 

Presenilins are the major genes responsible for familial Alzheimer’s disease (FAD). To elucidate the pathogenic mechanisms underlying FAD mutations, we employ advanced genetic approaches to investigate the normal function of presenilin in the brain and the dysfunction caused by disease causing mutations. Through development and multidisciplinary analysis of a number of cell type specific conditional mutant mice, we defined essential roles of presenilins in processes that are highly relevant to AD pathogenesis, such as memory, synaptic function and age-related neuronal survival. Our detailed analysis further elucidated the mechanisms by which presenilins regulate these processes, and whether presenilins regulate these processes *via* Notch receptors and in a γ-secretase-dependent manner. We also investigated the effects of various missense mutations in presenilin-1 associated with either FAD or frontotemporal dementia using both culture systems and knockin mice, and discovered their distinct molecular mechanisms. While FAD mutations cause partial to complete loss of γ-secretase activity *in cis*, mutant proteins further interfere the function of wild-type proteins *in trans.* The implications of our *in vivo* studies to disease mechanisms will be discussed.

